# Prognostic impact of right ventricular to pulmonary arterial coupling for patients undergoing surgery for severe mitral regurgitation

**DOI:** 10.1093/ehjimp/qyag068

**Published:** 2026-04-15

**Authors:** Roxana Botea, Yoan Lavie-Badie, Paul Bousquet, Francois Labaste, Etienne Grunenwald, Christophe Cron, Jean Porterie, Olivier Lairez, Bertrand Marcheix

**Affiliations:** Department of Cardiovascular Surgery, Rangueil University Hospital, Avenue Jean Poulhès, TSA 50032, Toulouse Cedex 31059, France; Department of Cardiology and Cardiovascular Medicine, Rangueil University Hospital, Toulouse, France; Department of Cardiology and Cardiovascular Medicine, Rangueil University Hospital, Toulouse, France; Department of Cardiovascular Surgery, Rangueil University Hospital, Avenue Jean Poulhès, TSA 50032, Toulouse Cedex 31059, France; Department of Anesthesiology and Intensive Care Unit, Rangueil University Hospital, Toulouse, France; Department of Cardiovascular Surgery, Rangueil University Hospital, Avenue Jean Poulhès, TSA 50032, Toulouse Cedex 31059, France; Department of Anesthesiology and Intensive Care Unit, Rangueil University Hospital, Toulouse, France; Department of Cardiovascular Surgery, Rangueil University Hospital, Avenue Jean Poulhès, TSA 50032, Toulouse Cedex 31059, France; Department of Cardiovascular Surgery, Rangueil University Hospital, Avenue Jean Poulhès, TSA 50032, Toulouse Cedex 31059, France; Department of Cardiovascular Surgery, Rangueil University Hospital, Avenue Jean Poulhès, TSA 50032, Toulouse Cedex 31059, France; Department of Cardiology and Cardiovascular Medicine, Rangueil University Hospital, Toulouse, France; Department of Cardiovascular Surgery, Rangueil University Hospital, Avenue Jean Poulhès, TSA 50032, Toulouse Cedex 31059, France

**Keywords:** RV-PA coupling, mitral surgery, mitral regurgitation

## Abstract

**Aims:**

Cardiac surgery may induce abrupt changes in pre-load, afterload, and right ventricular contractility. The ability of the right ventricle to maintain systolic performance in the presence of increased afterload is referred to as right ventricular–pulmonary artery (RV–PA) coupling. To assess RV–PA coupling in patients undergoing surgery for severe mitral regurgitation (MR), to identify a tricuspid annular plane systolic excursion to pulmonary artery systolic pressure (TAPSE/PASP) threshold for RV–PA uncoupling, and to evaluate its prognostic value for early mortality, right heart failure (RHF), post-operative course, and late mortality.

**Methods and results:**

This retrospective single-centre observational study included 277 patients who underwent surgery for severe MR between January 2018 and March 2023. RV–PA coupling was assessed using the ratio of TAPSE/PASP derived from pre-operative transthoracic echocardiography. The primary endpoint was early all-cause mortality within 30 days. Secondary endpoints included mortality from RHF, length of intensive care unit (ICU) stay, length of hospital stay, and late all-cause mortality.

Receiver operating characteristic analysis identified an optimal TAPSE/PASP cut-off of 0.30 mm/mmHg to define RV–PA uncoupling. Patients with TAPSE/PASP ≤0.30 mm/mmHg had significantly higher early mortality and worse post-operative outcomes. In multivariable logistic regression, TAPSE/PASP remained an independent predictor of early mortality together with EuroSCORE II, cardiopulmonary bypass time, and weaning from bypass requiring ECMO or inotropic support. In a sensitivity analysis restricted to isolated mitral valve (MV) surgery, TAPSE/PASP remained the only independent predictor of early mortality. RV–PA uncoupling was also associated with excess early mortality from RHF and longer ICU and hospital stay, but not with late mortality.

**Conclusion:**

Pre-operative RV–PA uncoupling assessed by TAPSE/PASP is strongly associated with early mortality and post-operative RHF after MV surgery for severe MR and may help refine perioperative risk stratification.

## Graphical Abstract

**Graphical Abstract:**
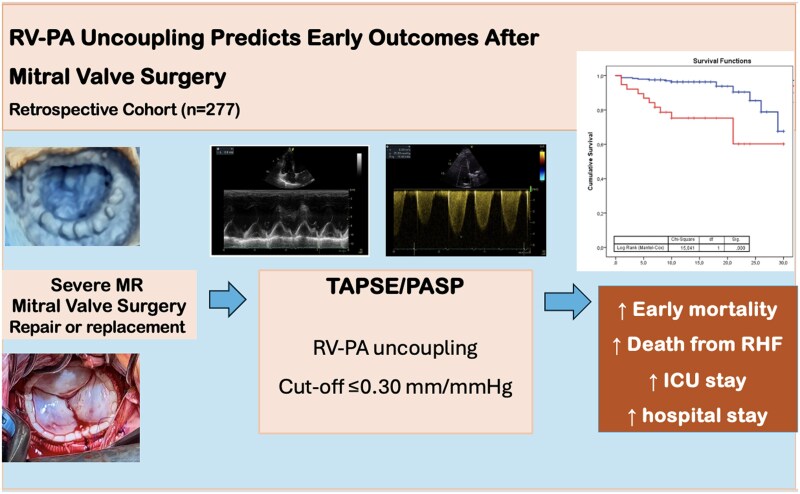

## Introduction

Despite its ubiquitous recognition as a crucial prognostic factor in cardiac surgery, right ventricular (RV) function remains underappreciated. Consequently, the most widely used cardiac surgical risk prediction scores—the Society of Thoracic Surgeons score and the European System for Cardiac Operative Risk Evaluation (EuroSCORE)—do not include indicators of RV function.^1,2^ Nevertheless, they do incorporate markers for pulmonary hypertension (PH). For what it’s worth, PH alone does not accurately reflect RV function in the context of mitral valve (MV) surgery.^3,4^

Earlier studies have reported a prevalence of nearly 30% for RV dysfunction (RVD) in the pre-operative population of patients with severe mitral regurgitation (MR).^3–5^ The primary pathophysiologic mechanism underlying RVD in severe MR involves persistently elevated pressure in the enlarged left atrium, with retrograde transmission to the pulmonary vasculature. This leads to progressively reduced pulmonary vascular compliance, ultimately increasing RV afterload and RV end-diastolic pressure. Chronic RV afterload initially triggers homeometric adaptation, in which RV contractility increases at the expense of diastolic function. When contractility can no longer be enhanced, heterometric adaptation occurs, characterized by RV dilation, tricuspid annular enlargement, valvular tenting, and tricuspid regurgitation (TR), establishing a vicious cycle of further RV volume overload and dysfunction.^6,7^

The RV’s ability to maintain systolic function in the face of increasing afterload is referred to as RV–pulmonary artery (PA) coupling.^8,9^ Coupling reflects the efficiency of energy transfer from the ventricle to the arterial load and describes the relationship between RV contractility and the afterload imposed by the pulmonary circulation.

Surgical procedures can induce abrupt changes in afterload or pre-load, or reduce RV contractility through mechanisms such as mechanical ventilation, pulmonary oedema, hypercapnia, acidosis, hypoxemia, systemic inflammatory response, intravenous fluid administration or transfusion, sepsis, arrhythmias, and coronary hypoperfusion or ischaemia.^10^

If increased afterload exceeds the RV’s capacity to augment contractility and maintain cardiac output, dysfunction or failure can develop, with a significant negative impact on surgical outcomes. RVD is common, affecting over 50% of hemodynamically unstable patients. RV failure (RVF), however, carries a markedly high mortality, ranging from 44% to 88% in various reports.^6,9,11–13^

The gold standard for characterizing RV–PA coupling involves invasive analysis of the volume–pressure loop, quantifying the ratio of end-systolic RV elastance to arterial elastance. Its invasiveness, however, limits widespread clinical use. We instead utilized a transthoracic echocardiography (TTE) parameter derived from the ratio of tricuspid annular plane systolic excursion (TAPSE) to pulmonary artery systolic pressure (PASP). TAPSE reflects RV longitudinal contractile function, and when indexed to PASP, it serves as a marker of contractile–pressure matching. A ratio below ∼0.31 mm/mmHg has been associated with RV–PA uncoupling and adverse outcomes in various populations, including patients with pulmonary arterial hypertension, heart failure with preserved or reduced ejection fraction, aortic stenosis, and severe MR undergoing surgical or percutaneous interventions.^14–26^

However, data specifically addressing the prognostic value of TAPSE/PASP for early post-operative mortality in a real-world cohort of patients undergoing surgery for severe MR remain limited. This study aims to address this gap by evaluating the predictive value of TAPSE/PASP in an unselected surgical population, thereby complementing and extending existing literature.

## Methods

### Study population

A systematic query of the surgical database of the Department of Cardiovascular Surgery at Toulouse Rangueil University Hospital was performed to identify patients who underwent surgery for severe MR between January 2018 and March 2023. Patients with severe mitral stenosis, congenital heart disease, or age <18 years were excluded. Redo cardiac surgery cases were also excluded because of the limited reliability of TAPSE measurements in this setting, as well as patients with massive TR, in whom PASP estimation may be underestimated due to right-sided pressure equalization. Patients without a complete pre-operative echocardiographic examination available for secondary analysis were additionally excluded.

Eligible patients underwent surgical MV repair or replacement for severe MR in accordance with contemporary guideline recommendations.^27^ Concomitant procedures—including tricuspid annuloplasty, surgical ablation for atrial fibrillation (AF) with or without left atrial appendage closure, aortic valve replacement, and coronary artery bypass grafting—were included. MR aetiology was predominantly degenerative, but also included functional, rheumatic, and infective causes. Both elective and urgent surgical cases were considered.

This retrospective, observational study based on routinely collected clinical data were approved by the institutional review board of Toulouse University Hospital (approval number RnPH 2025-25).

Baseline clinical characteristics included cardiovascular risk factors, comorbidities, New York Heart Association (NYHA) functional class, and pre-operative medical therapy.

### Echocardiographic data

RV–PA coupling parameters were derived from TTE examinations performed during the index hospitalization prior to surgical intervention. All studies were acquired at rest. Echocardiographic examinations were performed by the institutional core laboratory at Toulouse University Hospital using commercially available ultrasound systems (Vivid E95 and S70N, GE Vingmed) equipped with M5S transducers. Images were digitally stored in cine-loop format and subsequently analysed offline using dedicated software (EchoPAC version 203, GE Vingmed).

Image acquisition and analysis were conducted in accordance with the recommendations of the American Society of Echocardiography.^28^ TAPSE was measured using M-mode imaging with the cursor aligned with the direction of longitudinal RV shortening in the apical four-chamber view, from end-diastole to end-systole. In the same view, tricuspid lateral annular systolic velocity (*S*′ wave) was obtained using pulsed-wave tissue Doppler imaging.

PASP was estimated from the peak TR velocity obtained by continuous-wave Doppler, applying the simplified Bernoulli equation (PASP = 4 × *v*^2^ + right atrial pressure [RAP]). All patients included in the final study population had at least trivial TR, allowing for a reliable estimation of PASP and subsequent calculation of the TAPSE/PASP ratio. RAP was estimated based on inferior vena cava (IVC) diameter and respiratory collapsibility: an IVC diameter ≤21 mm with >50% inspiratory collapse suggested normal RAP (mean 3 mmHg, range 0–5 mmHg); an IVC diameter >21 mm with <50% collapse during a sniff maneuver indicated elevated RAP (mean 15 mmHg, range 10–20 mmHg); intermediate values were assigned in all other cases (mean 8 mmHg, range 5–10 mmHg).

RV–PA coupling was quantified using the TAPSE/PASP ratio. Left ventricular (LV) diameters were measured in the parasternal long-axis view at end-diastole and end-systole. LV and left atrial volumes were assessed using the biplane Simpson method and indexed to body surface area calculated using the Dubois formula. TR severity was graded using a multiparametric approach.

RV function was assessed according to American Society of Echocardiography recommendations, including longitudinal function (TAPSE and *S*′ wave), radial function (fractional area change), and loading conditions evaluated using qualitative and quantitative parameters (visual RV assessment, interatrial and interventricular septal position, basal RV-to-LV diameter ratio, and IVC diameter and collapsibility).^28^

### Endpoints

The primary clinical endpoint was early all-cause mortality, defined as death occurring within 30 days after surgery or during the index hospitalization, whichever occurred first.

Early right heart failure (RHF)-related mortality was assessed as a secondary endpoint. RHF was defined according to an inclusive definition derived from the Interagency Registry for Mechanically Assisted Circulatory Support, adapted to the perioperative cardiac surgery setting.^29^ RHF was characterized by persistent RVD with RAP >18 mmHg and cardiac index <2.0 L/min/m^2^, in the absence of elevated left-sided filling pressures (pulmonary capillary wedge pressure ≤18 mmHg), cardiac tamponade, sustained ventricular arrhythmias, or pneumothorax. RHF was considered present when these abnormalities required RV assist device implantation for failure to wean from cardiopulmonary bypass (CPB) or in the immediate post-operative period, and/or the use of inhaled nitric oxide and/or prolonged inotropic support.

Additional secondary endpoints included length of post-operative intensive care unit (ICU) stay, total in-hospital length of stay, and late all-cause mortality.

Late mortality was defined as death occurring more than 1 year after the index hospitalization. Vital status was ascertained retrospectively using Toulouse University Hospital medical records and, when necessary, by contacting patients’ general practitioners. Follow-up was completed on 25 September 2025.

### Statistical analysis

Continuous variables are presented as mean ± standard deviation or median with inter-quartile range (IQR), as appropriate. Categorical variables are expressed as absolute numbers and percentages. Normality of continuous variables was assessed using the Kolmogorov–Smirnov and Shapiro–Wilk tests. Comparisons between groups were performed using Student’s *t-*test for normally distributed variables and the Mann–Whitney *U* test for non-normally distributed variables. Categorical variables were compared using the χ^2^ or Fisher’s exact test, as appropriate.

Receiver operating characteristic (ROC) curve analysis was used to identify an optimal TAPSE/PASP cut-off for the discrimination of all-cause, early mortality. The optimal threshold was determined using the Youden index (TAPSE/PASP 0.30 mm/mmHg) Discriminatory performance was assessed by the area under the curve (AUC), with values >0.70 indicating acceptable/fair discrimination, >0.80 indicating good and >0.90 excellent discrimination.

Because deriving and testing a TAPSE/PASP cut-off within the same cohort may result in optimistic estimates of predictive performance, TAPSE/PASP was primarily analysed as a continuous variable in univariable and multivariable analyses. Given the short and fixed time horizon of the primary outcome (30-day mortality), logistic regression was selected as the most appropriate method to identify predictors of early all-cause mortality.

Multivariable logistic regression models were constructed with variable selection guided by clinical relevance and prior literature. To allow inclusion of a larger number of clinically meaningful covariates while minimizing the risk of overfitting, internal validation was performed using bootstrapping resampling with 2000 iterations. Collinearity among covariates was assessed using variance inflation factors.

To address the potential confounding effect related to procedural complexity, a secondary sensitivity analysis was performed after restricting the cohort to patients undergoing isolated MV surgery.

To explore the potential incremental discriminative value of TAPSE/PASP beyond established clinical and surgical risk factors, two multivariable logistic regression models were constructed: a first model including significant clinical and procedural predictors, and a second model additionally incorporating TAPSE/PASP. To visually illustrate the incremental discriminative effect of TAPSE/PASP, superimposed ROC curves derived from both models are presented. It is acknowledged that ROC curves reflect unadjusted discrimination and do not formally assess adjusted incremental predictive value.

Correlation between TAPSE and PASP was evaluated using Spearman’s rank correlation coefficient. Early and late cumulative survival was estimated using Kaplan–Meier curves, with differences between groups assessed using the Mantel–Cox log-rank test.

All statistical analyses were performed using SPSS software, version 23 (IBM Corp., Armonk, NY, USA). A two-sided *P-*value <0.05 was considered statistically significant.

## Results

**Figure 1.**
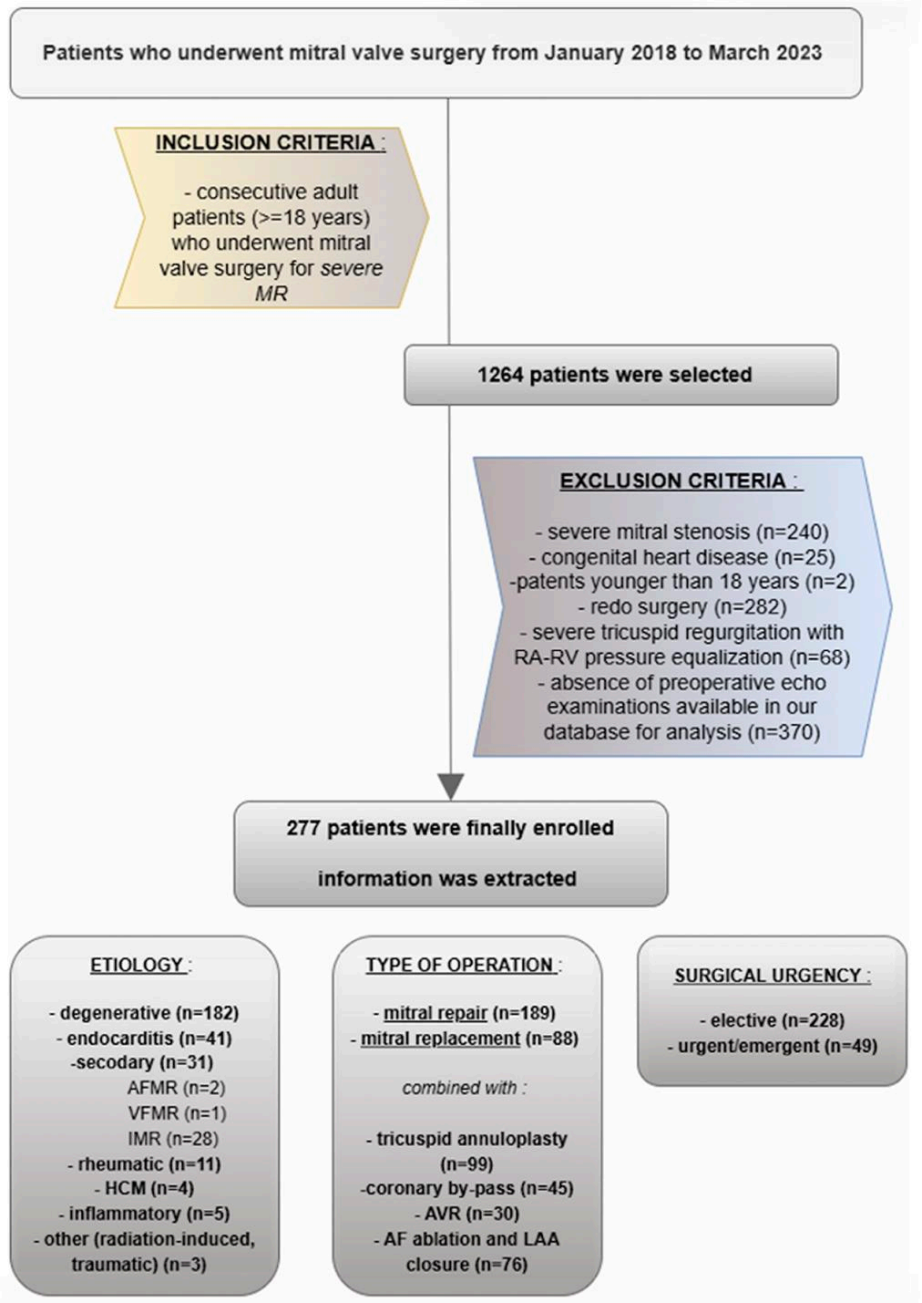
Flowchart of patient selection, exclusion criteria and final study cohort. AF, atrial fibrillation; AVR, aortic valve replacement; HCM, hypertrophic cardiomyopathy; LAA, left atrial appendicle; MR, mitral regurgitation; RA, right atrium; RV, right ventricle.

A total of 277 patients met the study inclusion criteria. Baseline clinical and echocardiographic characteristics of the overall cohort are summarized in *Table 1a*. The patient selection process, including applied inclusion and exclusion criteria, MR aetiologies, and derivation of the final study population, is detailed in *Figure 1*.

**Table 1:** Baseline, echocardiographic, procedural, and post-procedural parameters of general population

a	Overall cohort (*n* = 277)	TAPSE/PASP ≤0.3 mm/mmHg (*n* = 38)	TAPSE/PASP > 0.3 mm/mmHg (*n* = 239)	*P*-value
Age at intervention, y	63 ± 13	66 ± 15	63 ± 13	0.602
**M**ale sex	200 (72.2%)	24 (63.2%)	176 (73.6%)	0.122
**BMI,** kg/m**^**2**^**	1.86 ± 0.19	1.82 ± 0.2	1.87 ± 0.19	0.845
EuroSCORE **II**	**2.21** (**1.3–3.7)**	**4.48** (**3.1–5.75)**	**1.86** (**1.24–3.21)**	**<0**.**001**
Hypertension	118 (42.6%)	19 (50%)	99 (41.4%)	0.243
Ibrillation	**96** (**34.7%)**	**20** (**52.6%)**	**76** (**31.8%)**	**0**.**011**
**COPD**	24 (8.7%)	4 (10.5%)	20 (8.4%)	0.423
Coronary artery disease	**74** (**26.7%)**	**19** (**50%)**	**55** (**23%)**	**<0**.**001**
Diabetes	18 (6.5%)	2 (5.3%)	16 (6.7%)	0.543
**GFR (**mL**/**min**/1.73 **cm^**3**^**)**	74 ± 22	61 ± 22	76 ± 22	0.665
baseline functional **NYHA** class **III/IV**	**129** (**46.6%)**	**29** (**76.3%)**	**100** (**41.8%)**	**<0**.**001**
Peripheral oedema at presentation	**75** (**27.1%)**	**21** (**55.3%)**	**54** (**22.6%)**	**<0**.**001**
Echocardiographic parameters				
**LVED** diameter (mm)	**55** **±** **7**	**56** **±** **9**	**55** **±** **7**	**0**.**021**
**LVES** diameter (mm**)**	**38** **±** **7**	**40** **±** **9.3**	**37** **±** **7**	**0**.**044**
**LVED** volume **(**mL**/**m^**2**^**)**	**74** **±** **24**	**80** **±** **30**	**73** **±** **22**	**0**.**002**
**LVEF %**	62 (56–67)	58 (44–65)	62 (57–57)	0.071
**LA** volume	62 ± 31	71 ± 32	60 ± 31	0.312
***S*´**	13 ± 3	10 ± 3	13 ± 3	0.789
**TAPSE (**mm**)**	22 ± 5	16 ± 4	23 ± 4	0.922
**PASP (**mmHg**)**	**41** **±** **16**	**64** **±** **19**	**38** **±** **11**	**<0**.**001**
**RAP (**mmHg**)**	**5** (**5–5)**	**6.5** (**5–10)**	**5** (**5–5)**	**0**.**001**
**TAPSE/PASP (**mm/mm**H**g**)**	**0.6** **±** **0.25**	**0.25** **±** **0.04**	**0.66** **±** **0.23**	**<0**.**001**
Tricuspid annulus (mm**)**	**38** (**34–42)**	**41** (**37–44)**	**37** (**34–41)**	**<0**.**001**
**TR** mild	**235** (**84.8%)**	**22** (**57.9%)**	**213** (**89.1%)**	**<0**.**001**
**TR** moderate	**30** (**10.8%)**	**15** (**39.5%)**	**15** (**6.3%)**	**<0**.**001**
**TR** severe	6 (2.2%)	1 (2.6%)	5 (2.1%)	0.594
**A**etiology				
**S**evere aortic stenosis	7 (2.5%)	0 (0%)	7 (2.9%)	0.356
Severe aortic insufficiency	12 (4.3%)	2 (5.3%)	10 (4.2%)	0.528
Degenerative aetiology	182 (65.7%)	20 (52.6%)	162 (67.8%)	0.052
Rheumatic aetiology	**11** (**4%)**	**4** (**10.5%)**	**7** (**2.9%)**	**0**.**049**
Endocarditis	41 (14.9%)	5 (13.5%)	36 (15.1%)	0.516
Secondary (ischaemic, atrial)	31 (11.2%)	7 (18.4%)	24 (10%)	0.114
**b** Procedural parameters				
Urgent surgery	49 (17.7%)	7 (18.4%)	42 (17.6%)	0.524
Mitral replacement vs. mitral repair	**88** (**31.8%)**	**18** (**47.4%)**	**70** (**29.3%)**	**0**.**027**
Aortic replacement	31 (11.2%)	3 (7.9%)	28 (11.7%)	0.358
Tricuspid annuloplasty	**99** (**35.7%)**	**22** (**57.9%)**	**77** (**32.2%)**	**0**.**002**
Coronary bypass	**45** (**16.2%)**	**11** (**28.9%)**	**34** (**14.2%)**	**0**.**025**
**AF** ablation **± LAA** closure	76 (27.4%)	14 (36.8%)	62 (25.9%)	0.115
Combined surgery	**184** (**66.4%)**	**31** (**81.6%)**	**153** (**64%)**	**0**.**023**
Aortic cross-clamp time (min**)**	70 (55–94)	78 (52–91)	69n (56–94)	0.973
**CPB** time (min)	92 (74–123)	99 (74–120)	91 (74–123)	0.594
Post**-**procedural outcomes and follow-up				
Weaning with inotropes	**79** (**28.5%)**	**27** (**71.1%)**	**52** (**21.8%)**	**<0**.**001**
Weaning with **ECMO**	**9** (**3.2%)**	**6** (**15.8%)**	**3** (**1.3%)**	**<0**.**001**
Right heart failure	**31** (**11.2%)**	**15** (**39.5%)**	**16** (**6.7%)**	**<0**.**001**
Right heart dysfunction	**89** (**32.1%)**	**24** (**63.2%)**	**65** (**27.2%)**	**<0**.**001**
**ICU** stay (days)	**5** **±** **6**	**8** **±** **10**	**5** **±** **5**	**0**.**009**
Ventilation (days)	**1.4** **±** **6**	**3.9** **±** **10**	**1** **±** **5**	**<0**.**001**
**KDIGO**	**0** (**0–1)**	**1** (**0–3)**	**0** (**0–0)**	**<0**.**001**
Inotrope administration time (days)	**0** (**0–1)**	**2** (**0.7–5)**	**0** (**0–0)**	**0**.**001**
Hospital stay (days)	**13** **±** **8**	**16** **±** **14**	**12** **±** **7**	**<0**.**001**
**LVEF%** at discharge	55 (49–60)	50 (36–58)	55 (50–60)	0.087
Furosemide dose at discharge (mg)	**40** (**0–80)**	**80** (**0–122)**	**40** (**0–80)**	**0**.**032**
All cause early mortality	**24** (**8.7%)**	**11** (**28.9%)**	**13** (**5.4%)**	**<0**.**001**
Early mortality of **RHF**	**14** (**5.1%)**	**9** (**23.7%)**	**5** (**2.1%)**	**<0**.**001**
All cause late mortality	12/253 (4.7%)	1/27 (3.7%)	11 (4.9%)	0.626

BMI, body mass index; COPD, chronic obstructive pulmonary disease; CPB, cardiopulmonary bypass; ECMO, extracorporeal membrane oxygenation; GFR, glomerular filtration rate; ICU, intensive care unit; KDIGO, kidney disease: improving global outcomes; LVED, left ventricular end-diastolic; LVES, left ventricular end systolic; LA, left atrial; LAA, left atrial appendicle; LVEF, left ventricular ejection fraction; TAPSE, tricuspid annular plane systolic excursion; PASP, pulmonary artery systolic pressure; NYHA, New York Heart Association; RAP, right atrial pressure; TR, tricuspid regurgitation.

Values which are continuous variables are reported as mean ± SD or median (IQR). Bold values indicate statistical significance (*P* < 0.05).

The majority of patients had degenerative MR (*n* = 182, 65.7%), followed by endocarditis (*n* = 41, 14.9%), secondary MR (SMR) (*n* = 31, 11.2%), including atrial functional (*n* = 2), ventricular functional (*n* = 1), and ischaemic (*n* = 28) aetiologies, and rheumatic disease (*n* = 11, 4%). Most patients exhibited preserved or mildly reduced LV ejection fraction (median 62%, IQR 56–67) and mild TR (*n* = 235, 84.8%).

### RV–PA uncoupling

**Figure 2.**
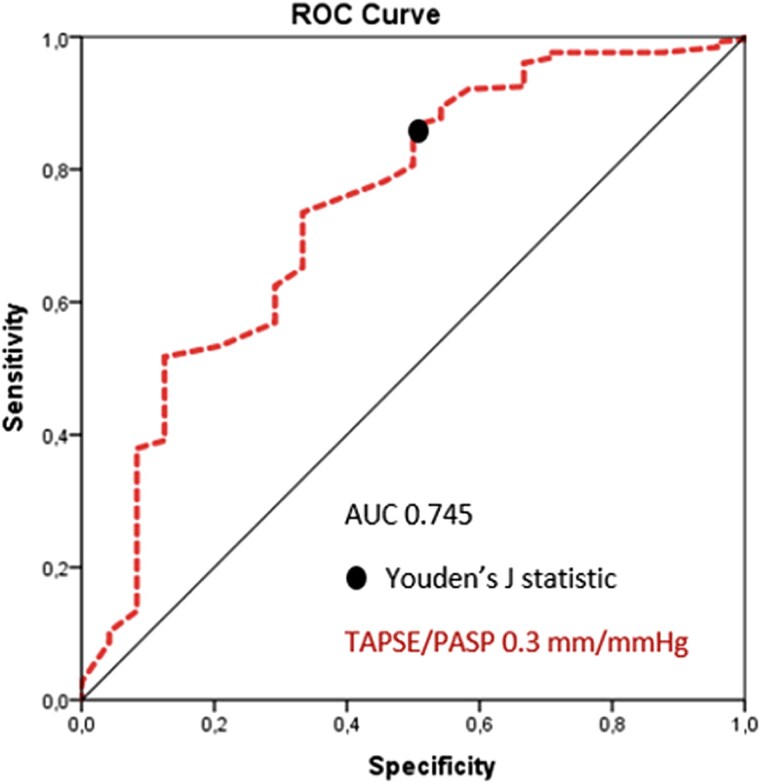
Receiver operating characteristic curve for all-cause early mortality.

Across the study population, the mean TAPSE/PASP ratio was 0.61 ± 0.25 mm/mmHg. ROC analysis indicated acceptable discrimination for early all-cause mortality (AUC 0.745, *Figure 2*), with a cut-off of 0.30 mm/mmHg defining RV–PA uncoupled (≤0.30) and coupled (>0.30) patients.

RV–PA uncoupled patients were more symptomatic (NYHA III/IV: 76.3% vs. 41.8%, *P* < 0.001), had higher prevalence of coronary artery disease (50% vs. 23%, *P* < 0.001) and AF (52.6% vs. 31.8%, *P* < 0.001), and higher surgical risk (EuroSCORE II: 4.48 vs. 1.86, *P* < 0.001). Echocardiography showed larger LV end-systolic diameter, higher prevalence of moderate TR, more dilated tricuspid annuli, and elevated PASP, while TAPSE was similar.

Rheumatic MR was associated with more frequent uncoupling; other aetiologies showed no differences. Available MV data showed mean valve areas of 2.6 cm^2^ in rheumatic MR and 4.2 cm^2^ in degenerative MR undergoing replacement.

Procedurally, uncoupled patients underwent more frequent MV replacement (47.4% vs. 29.3%, *P* = 0.027), combined surgeries (81.6% vs. 64%, *P* = 0.023), tricuspid annuloplasty (57.9% vs. 32.2%, *P* = 0.002), and concomitant coronary artery bypass grafting (CABG) (28.9% vs. 14.2%, *P* = 0.025).

In the isolated mitral surgery subgroup, RV–PA uncoupled patients were more symptomatic, had more comorbidities, and higher EuroSCORE II, while aetiology, type of intervention, and CPB time (72 [61–86] min) did not differ (*Table 4*).

### RV–PA uncoupling and outcomes

Among 277 patients, 24 (8.7%) experienced early, in-hospital all-cause death, including 14 (5.1%) from RHF (*Table 1b*). *Table 2* presents early mortality according to MR aetiology. Inflammatory (lupus and anorexigenic-associated) and SMR showed the highest proportional mortality, mainly due to biventricular failure. Degenerative MR accounted for most early deaths, including cases of RHF (*n* = 5), biventricular failure (*n* = 3), tamponade, bleeding, mitral annulus rupture, and septic shock; detailed causes are provided in the table.

**Table 2:** Mortality stratified by MRs aetiology

MR aetiology (*n* = 277)	Early death (*n* = 23)	Cause
Degenerative (*n* = 182)	***n*** **=** **12 (6.5%)**	
	*n* = 5	RHF
	*n* = 3	BiVF
	*n* = 1	Tamponade
	*n* = 1	Bleeding
	*n* = 1	Mitral annulus rupture
	*n* = 1	Acute pancreatitis and septic choc
Rheumatic (*n* = 11)		
Endocarditis (*n* = 41)	***n*** **=** **3 (7.3%)**	
	*n* = 1	Septic choc
	*n* = 1	BiVF
	*n* = 1	Respiratory compromise
Inflammatory (*n* = 5)	***n*** **=** **2 (40%)**	
	*n* = 1	LHF
	*n* = 1	BiVF
Secondary (*n* = 31)	***n*** **=** **7 (22.5%)**	
	*n* = 4	BiVF
	*n* = 2	RHF
	*n* = 1	Bleeding
HCM (*n* = 4)		
Other (radiation induced, traumatic) (*n* = 3)		

BiVF, biventricular heart failure; LVF, left ventricular heart failure; RHF, right heart failure. Bold values represent subgroup totals and are presented for visual clarity only, without implying statistical significance.

In the RV–PA uncoupled group, all-cause mortality was higher than in coupled patients (29% vs. 5.4%, *P* < 0.001), as was RHF-related death (23% vs. 2.1%, *P* < 0.001).

**Figure 3.**
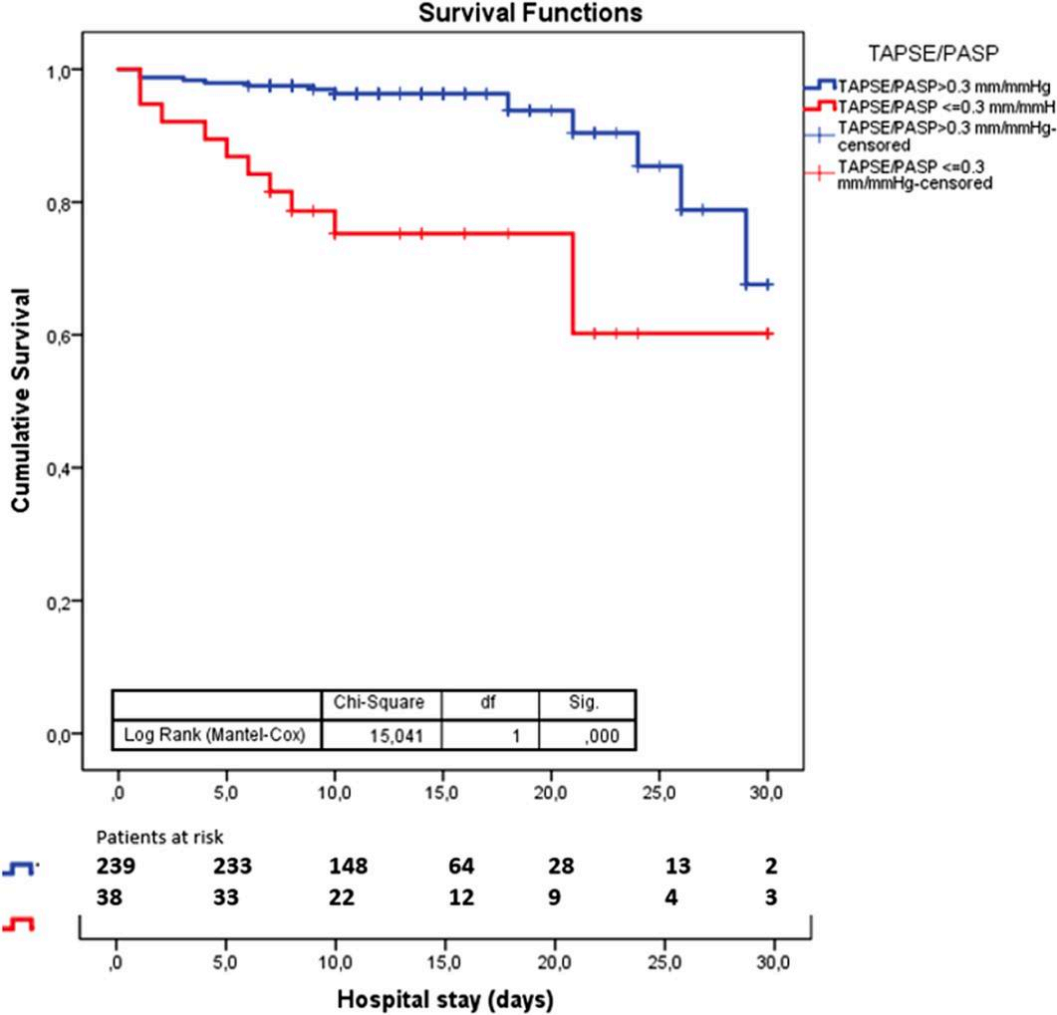
Kaplan Meier curves for all-cause early mortality stratified by TAPSE/PASP subgroups.

Kaplan–Meier analysis confirmed significantly elevated early mortality in uncoupled patients (log-rank χ^2^ 15.041, *P* < 0.001; *Figure 3*).

In univariable logistic regression analysis of the overall cohort, age, EuroSCORE II, AF, baseline NYHA functional class, LVEF, TAPSE/PASP, PASP >50 mmHg, SMR, MV replacement, as well as combined surgical procedures, concomitant CABG, CPB and aortic cross-clamp times, and post-procedural need for CPB weaning with ECMO or inotropic support were all significantly associated with early (30-day) all-cause mortality. In the multivariable model, only EuroSCORE II, TAPSE/PASP, CPB time, and weaning from CPB with ECMO remained statistically significant predictors (*Table 3*).

**Table 3:** Logistic regression analysis of clinical, echocardiographical and procedural predictors of early all-cause mortality for general population

	Univariable		Multivariable	
	OR (95% CI)	*P*-value	OR (95% CI)	*P*-value
Age at intervention, y	**1.042** (**1.002–1.083)**	**0**.**040**		
Male sex	1.63 (0.683–3.904)	0.271		
BMI, kg/m**^**2**^**	0.165 (0.17–1.609)	0.121		
EuroSCORE II	**1.279** (**1.146–1.427)**	**<0**.**001**	**1.221** (**1.061–1.406)**	**0**.**005**
Hypertension	1.155 (0.498–2.677)	0.866		
Atrial fibrillation	**4.325** (**1.778–10.552)**	**0**.**001**	2.301 (0.819–6.459)	0.114
COPD	**5.716** (**2.085–15.670)**	**0**.**001**		
Coronary artery disease	**4.503** (**1.902–10.650)**	**0**.**001**		
Diabetes	1.347 (0.291–3.442)	0.684		
GFR (mL/min/1.73 cm^3^)	**0.963** (**0.945–0.980)**	**<0**.**001**		
Baseline functional NYHA class III/IV	**2.478** (**1.025–5.999)**	**0**.**044**		
Peripheral oedema at presentation	**2.499** (**1.066–5.855)**	**0**.**035**		
Echocardiographic parameters				
LVED diameter (mm)	0.997 (0.942–1.055)	0.916		
LVES diameter (mm)	1.014 (0.951–1.082)	0.669		
LVED volume **(**mL**/**m^2^)	1.006 (0.990–1.023)	0.449		
LVEF %	**0.962** (**0.932–0.992)**	**0**.**015**		
LA volume	1.005 (0.995–1.016)	0.332		
***S´***	**0.780** (**0.655–0.928)**	**0**.**005**		
TAPSE (mm)	**0.880** (**0.809–0.957)**	**0**.**003**		
PASP (mmHg)	**1.038** (**1.015–1.061)**	**0**.**001**		
RAP (mmHg)	**1.148** (**1.018–1.295)**	**0**.**024**		
TAPSE/PASP (mm/mmHg)	**0.015** (**0.002–0.148)**	**<0**.**001**	**0.039** (**0.004–0.635)**	**0**.**021**
PASP > 50 mmHg	**2.722** (**1.159–6.391)**	**0**.**022**		
Tricuspid annulus (mm)	0.970 (0.894–1.054)	0.475		
TR mild	**0.203** (**0.083–0.495)**	**<0**.**001**		
TR moderate	**6.629** (**2.591–16.958)**	**<0**.**001**		
TR severe	0.464 (0.52–4.140)	0.491		
Aetiology				
Degenerative aetiology	2.048 (0.882–4.754)	0.095		
Endocarditis	1.178 (0.334–4.160)	0.799		
Secondary (ischaemic, atrial)	**3.929** (**1.481–10.421)**	**0**.**005**		
Procedural parameters				
Urgent surgery	2.069 (0.808–5.298)	0.130	0.756 (0.186–3.070)	0.696
Mitral replacement vs. Mitral repair	**3.386** (**1.432–7.961)**	**0**.**005**	2.271 (0.872–5.915)	0.093
Aortic replacement	1.148 (0.322–4.095)	0.832		
Tricuspid annuloplasty	1.123 (0.463–2.727)	0.797	0.616 (0.227–1.673)	0.342
Coronary bypass	**3.617** (**1.473–8.882)**	**0**.**005**		
AF ablation ± LAA closure	1.93 (0.84–4.4)	0.115		
Combined surgery	**3.865** (**1.122–13.313)**	**0**.**032**	0.991 (0.186–3.070)	0.990
Aortic cross-clamp time **(**min**)**	**1.012** (**1.001–1.023)**	**0**.**029**		
CPB time (min)	**1.014** (**1.005–1.024)**	**0**.**003**	**1.103** (**1.002–1.024)**	**0**.**021**
Post**-**procedural outcomes and follow-up				
Weaning with inotropes	**6.032** (**2.464–14.765)**	**<0**.**001**	2.314 (0.770–6.961)	0.135
Weaning with ECMO	**27.778** (**6.412–120.344)**	**<0**.**001**	**9.018** (**1.873–43.416)**	**0**.**006**
ICU stay (days)	**1.078** (**1.024–1.35)**	**0**.**004**		
Ventilation (days)	**1.205** (**1.086–1.338)**	**<0**.**001**		
KDIGO	**5.719** (**3.360–9.735)**	**<0**.**001**		
Inotrope administration time (days)	**1.409** (**1.234–1.609)**	**<0**.**001**		

COPD, chronic obstructive pulmonary disease; CPB, cardiopulmonary bypass; LVEF, left ventricular ejection fraction; TAPSE, tricuspid annular plane systolic excursion; PASP, pulmonary artery systolic pressure; NYHA, New York Heart Association; RAP, right atrial pressure; TR, tricuspid regurgitation.

Bold values indicate statistical significance (*P* < 0.05).

When the analysis was restricted to patients undergoing isolated MV surgery, post-procedural outcomes and early all-cause and RHF mortality were significantly higher in the uncoupled group (*Table 4*). A multivariable logistic regression with bootstrapping (2000 resamples) was performed to account for the limited number of events. In this subset, TAPSE/PASP remained independently associated with early all-cause mortality (BCa 95% CI: −605.21 to −3.89, *P* = 0.001), whereas EuroSCORE II was no longer statistically significant (BCa 95% CI: −10.99 to 6.20, *P* = 0.367) (*Table 5*).

**Table 4:** Univariable comparison of baseline clinical, echocardiographic, and procedural characteristics between RV–PA uncoupled and RV–PA coupled patients in the subgroup undergoing isolated MV surgery

	Isolated MR (n = 93)	TAPSE/PASP ≤0.3 mm/mmHg (*N* = 7)	TAPSE/PASP > 0.3 mm/mmHg (*N* = 86)	*P*
Age at intervention, y	65 ± 12	56 ± 25	55 ± 14	0.955
Male sex	57 (61.3%)	4 (57.1%)	53 (61.6%)	0.556
EuroSCORE II	**1.2** (**0.8–2.2)**	**4** (**2.2–5.1)**	**1.2** (**0.7–1.9)**	**<0**.**001**
Atrial fibrillation	6 (6.5%)	2 (28.6%)	4 (4.7%)	0.063
GFR (mL/min/1.73 cm^3^)	**78** **±** **24**	**57** **±** **26**	**79** **±** **24**	**0**.**024**
Baseline functional NYHA class III/IV	**36** (**38.7%)**	**7** (**100%)**	**29** (**33.7%)**	**0**.**001**
Peripheral oedema at presentation	**22** (**23.7%)**	**4** (**57.1%)**	**18** (**120.9%)**	**0**.**052**
Echocardiographic parameters				
LVED diameter (mm)	54 ± 7	59 ± 11	54 ± 6	0.096
LVES diameter (mm)	**36** **±** **7**	**43** **±** **10**	**36** **±** **6**	**0**.**017**
LVED volume (mL/m^2^)	71 ± 20	82 ± 30	74 ± 22	0.007
LVEF %	**63** **±** **8**	**53** **±** **17**	**64** **±** **7**	**0**.**002**
LA volume	54 ± 19	61 ± 15	54 ± 19	0.380
*S´*	**14** **±** **3**	**10** **±** **2**	**14** **±** **3.8**	**0**.**008**
TAPSE (mm)	**24** **±** **4**	**17** **±** **4**	**24** **±** **5**	**0**.**001**
PAPS (mmHg)	**38** **±** **16**	**77** **±** **21**	**35** **±** **11**	**<0**.**001**
RAP (mmHg)	**5** (**5–5)**	**10** (**5–15)**	**5** (**5–5)**	**<0**.**001**
TAPSE/PAPS (mm/mmHg)	**0.7** **±** **0.24**	**0.22** **±** **0.05**	**0.74** **±** **0.23**	**<0**.**001**
TR mild	**87** (**93.5%)**	**4** (**57.1%)**	**83** (**96.5%)**	**0**.**005**
TR moderate	**4** (**4.3%)**	**3** (**42.9%)**	**1** (**1.2%)**	**0**.**001**
Aetiology				
Degenerative	58 (62.4%)	4 (57.1%)	54 (62.8%)	0.531
Rheumatic	1 (1.1%)	0 (0%)	1 (1.2%)	0.925
Endocarditis	22 (23.9%)	1 (16.7%)	21 (24.4%)	0.557
Secondary	5 (5.4%)	1 (14.3%)	4 (4.7%)	0.330
Procedural parameters				
Urgent surgery	23 (24.7%)	3 (43.9%)	20 (23.3%)	0.232
Mitral repair vs. Mitral replacement	67 (72%)	3 (42.9%)	64 (74.4%)	0.093
Aortic cross-clamp time (min)	55 (45–65)	45 (32–73)	55 (46–65)	0.370
CPB time (min)	72 (61–86)	72 (55–99)	73 (61–86)	0.827
Post-procedural outcomes and follow-up				
Weaning with inotropes	**12** (**12.9%)**	**5** (**71.4%)**	**7** (**8.1%)**	**<0**.**001**
Weaning with ECMO	**2** (**2.2%)**	**2** (**28.6%)**	**0** (**0%)**	**0**.**005**
Right heart failure	**4** (**4.3%)**	**2** (**28.6%)**	**2** (**2.3%)**	**0**.**027**
Right heart dysfunction	**22** (**23.7%)**	**5** (**71.4%)**	**17** (**19.8%)**	**0**.**007**
ICU stay (days)	**4** **±** **5**	**10** **±** **13**	**3** **±** **3**	**0**.**002**
Ventilation (days)	**1** **±** **5**	**4** **±** **11**	**1** **±** **6**	**0**.**039**
KDIGO	**0** (**0–0)**	**3** (**0–3)**	**0** (**0–0)**	**0**.**001**
Inotrope administration time (days)	**0** (**0–0)**	**1** (**0–7)**	**0** (**0–0)**	**0**.**001**
Hospital stay (days)	**11** **±** **6**	15 ± 16	11 ± 5	0.131
LVEF% at discharge	65 (60–68)	46 (38–62)	55 (50–60)	0.313
Furosemide dose at discharge (mg)	40 (40–40)	122 (75–125)	40 (40–80)	0.121
All cause early mortality	**3** (**3.2%)**	**2** (**28.6%)**	**1** (**1.2%)**	**0**.**014**
Early mortality of RHF	**2** (**2.2%)**	**2** (**28.6%)**	**0** (**0%)**	**0**.**005**
All cause late mortality	4 (4.4%)	1 (20%)	3 (3.5%)	0.208

BMI, body mass index; COPD, chronic obstructive pulmonary disease; CPB, cardiopulmonary bypass; ECMO, extracorporeal membrane oxygenation; GFR, glomerular filtration rate; ICU, intensive care unit; KDIGO, kidney disease: improving global outcomes; LVED, left ventricular end-diastolic; LVES, left ventricular end systolic; LA, left atrial; LAA, left atrial appendicle; LVEF, left ventricular ejection fraction; TAPSE, tricuspid annular plane systolic excursion; PASP, pulmonary artery systolic pressure; NYHA, New York Heart Association; RAP, right atrial pressure; TR, tricuspid regurgitation.

Values which are continuous variables are reported as mean ± SD or median (IQR). Bold values indicate statistical significance (*P* < 0.05).

**Table 5:** Univariable and multivariable logistic regression analysis for early all-cause mortality for isolated MR population

	Univariable		Multivariable	
	OR (95% CI)	*P*-value	OR (95% CI)	*P*-value
Age at intervention, y	0.988 (0.918–1.062)	0.740		
Male sex	1.273 (0.111–14.567)	0.846		
**E**uroSCORE II	**1.599** (**1.070–2.390)**	**0**.**022**	1.05 (−10.99 to 6.20)	0.367
Atrial fibrillation	8.5 (0.654–112.429)^a^	0.102		
GFR (mL/min/1.73 cm^3^)	0.974 (0.934–1.016)	0.226		
Baseline functional NYHA class III/IV	3.294 (0.228–37.717)	0.338		
peripheral oedema at presentation	1.693 (1.142–19.031)	0.691		
Echocardiographic parameters				
LVES diameter (mm)	1.152 (0.971–1.368)	0.104		
LVED volume (mL/m^2^)	1.036 (0.986–1.089)	0.158		
LVEF %	**0.883** (**0.807–0.967)**	**0**.**007**		
LA volume	**1.046** (**1.006–1.087)**	**0**.**024**		
TAPSE (mm)	**0.766** (**0.588–0.998)**	**0**.**048**		
PASP (mmHg)	**1.061** (**1.012–1.112)**	**0**.**014**		
RAP (mmHg)	**1.308** (**1.032–1.658)**	**0**.**026**		
TAPSE/PASP (mm/mmHg)	**<0.01** (**0–0.463)**^a^	**0**.**033**	**0.02 (−605.21 to −3.89)**	**0**.**01**
PASP > 50 mmHg	9.259 (0.790–105.327)^a^	0.076		
Aetiology				
Degenerative aetiology	0.289 (0.025–3.316)	0.319		
Secondary (ischaemic, atrial)	10.750 (0.797–140.936)^a^	0.075		
Procedural parameters				
Urgent surgery	1.545 (0.134–170.876)^a^	0.727		
Mitral replacement vs. mitral repair	5.500 (0.477–53.450)^a^	0.172		
Aortic cross-clamp time (min)	0.966 (0.888–1.051)	0.422		
CPB time (min)	1.004 (0.958–1.052)	0.875		
Post-procedural outcomes and follow-up				
Weaning with inotropes	**16** (**1.328–192.758)**^a^	**0**.**029**		
Weaning with ECMO	**44.5** (**1.993–993.344)**^a^	**0**.**017**		
ICU stay (days)	**1.112** (**1.003–1.232)**	**0**.**044**		
Ventilation (days)	**1.881** (**1.133–3.125)**	**0**.**015**		
KDIGO	**5.817** (**1.421–23.806)**	**0**.**014**		
Inotrope administration time (days)	**1.546** (**1.059–2.259)**	**0**.**024**		

Odds ratios and 95% bias-corrected and accelerated (BCa) confidence intervals were derived using bootstrapping with 2000 resamples to account for the limited number of events. TAPSE/PASP remained independently associated with early mortality, whereas EuroSCORE II did not.

^a^Estimates should be interpreted with caution due to sparse data.

CPB, cardiopulmonary bypass; ECMO, extracorporeal membrane oxygenation; GFR, glomerular filtration rate; ICU, intensive care unit; KDIGO, kidney disease: improving global outcomes; LVED, left ventricular end-diastolic; LVES, left ventricular end systolic; LA, left atrial; LAA, left atrial appendicle; LVEF, left ventricular ejection fraction; TAPSE, tricuspid annular plane systolic excursion; PASP, pulmonary artery systolic pressure; NYHA, New York Heart Association; RAP, right atrial pressure. Bold values indicate statistical significance (*P* < 0.05).

**Figure 4.**
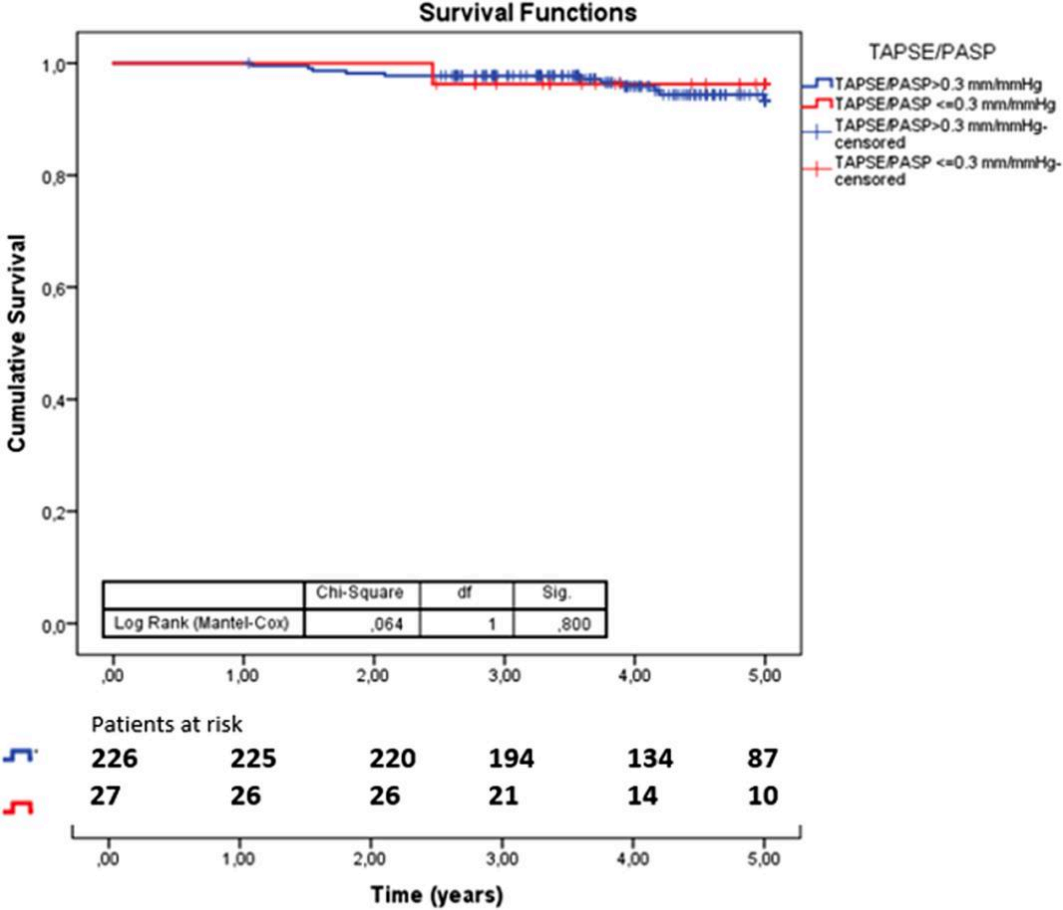
Kaplan Meier curves for all-cause late mortality stratified by TAPSE/PASP subgroups

Uncoupled patients had longer ICU (median 5.5 vs. 4 days, *P* < 0.001) and hospital stays (median 10.5 vs. 11 days, *P* < 0.001) in the general population. During a median follow-up of 4.4 years, 12 deaths (4.7%) occurred, with no significant difference in late mortality between groups (*Figure 4*).

## Discussion

This study non-invasively evaluated RV–PA coupling in patients with severe MR undergoing MV surgery. The main finding is that RV–PA coupling, assessed by TAPSE/PASP, is a strong and independent predictor of early all-cause mortality, including death from RHF. Moreover, RV–PA uncoupling is associated with worse post-procedural outcomes, including prolonged ICU and hospital stays.

Current guidelines for MV surgery primarily focus on symptoms and LV morphology and function, with RV function not being a primary consideration.^27^ Secondary criteria such as PH (PASP >50 mmHg) or tricuspid annulus dilation ± TR reflect the effects of chronic MR on the pulmonary circulation and RV through long-standing retrograde volume and pressure overload.^30^ Early studies have characterized the RV response to chronic pressure overload, describing an initial adaptive (homeometric) phase followed by maladaptive (heterometric) remodeling.^7,30,31^ The transition to maladaptation is marked by RV exhaustion and an insufficient contractile response to increased afterload, defining the ‘uncoupled’ RV–PA state. Cardiac surgery represents a unique setting in which abrupt changes in pre-load and afterload challenge the RV at this maladaptive stage, when contractility can no longer compensate.

This study demonstrates that RV–PA uncoupling, assessed by TAPSE/PASP, is a robust predictor of early mortality in patients undergoing mitral surgery. Mechanistically, uncoupling reflects the RV’s inability to adapt to abrupt hemodynamic stress, as occurs during surgery in the context of chronic pressure and volume overload. In the overall cohort, TAPSE/PASP was strongly associated with 30-day all-cause mortality in both univariable and multivariable analyses, alongside factors such as combined procedures and prolonged CPB time, which may act cumulatively to precipitate RHF and death. To account for potential confounding by procedural complexity, we analysed the isolated mitral surgery subgroup separately. Here, univariable analysis confirmed a strong association between TAPSE/PASP and early mortality, together with EuroSCORE, RAP, LVEF, and LA volume, while CPB time lost significance. Importantly, multivariable analysis in this subgroup identified TAPSE/PASP as the sole independent predictor of 30-day mortality, highlighting its incremental and specific prognostic value in the context of RV adaptation to surgical stress.

Previous studies have investigated the prognostic role of TAPSE/PASP in severe MR.^16–18^ Rzucidlo-Resil *et al*. identified a cut-off of <0.42 mm/mmHg for early mortality after surgery for SMR, slightly higher than the <0.30 mm/mmHg threshold observed in our cohort.^17^ In our SMR subgroup, no significant association with RV–PA uncoupling was found, although 30-day mortality was higher, likely reflecting LV disease rather than RV–PA impairment; the small sample (*n* = 31) limits definitive conclusions.

Chehab *et al*. reported a cut-off of ∼0.30 mm/mmHg, closely matching our overall cohort.^17^ Their analysis, however, excluded endocarditis and combined procedures, while our study reflects a real-life population. Furthermore, complementing Chehab *et al*.’s work, who focused on late mortality, our study highlights the immediate post-operative period, when RV–PA uncoupling is also impactful, demonstrated by the significant early mortality and RHF in uncoupled patients. In contrast, long-term outcomes did not differ between coupled and uncoupled groups, likely because mitral surgery—particularly physiologic repair or prosthetic replacement without residual transvalvular gradient—effectively reduces PH.^32,33^

Importantly, a recent systematic review and meta-analysis by Androshchuk *et al*. identified an optimal TAPSE/PASP cut-off of ∼0.33 mm/mmHg for predicting all-cause mortality (both early and late) and major adverse cardiovascular events in patients undergoing MV interventions.^18^ In our surgical cohort, the optimal threshold for predicting early mortality due to RHF was 0.30 mm/mmHg, which is remarkably consistent with the value reported in the literature. This concordance reinforces the robustness of the TAPSE/PASP ratio as a marker of RV–PA uncoupling across different clinical contexts, including both interventional and surgical MV procedures. Minor differences in the optimal cut-off may reflect variations in patient characteristics, disease severity, and the timing of outcome assessment between study populations.

**Figure 5.**
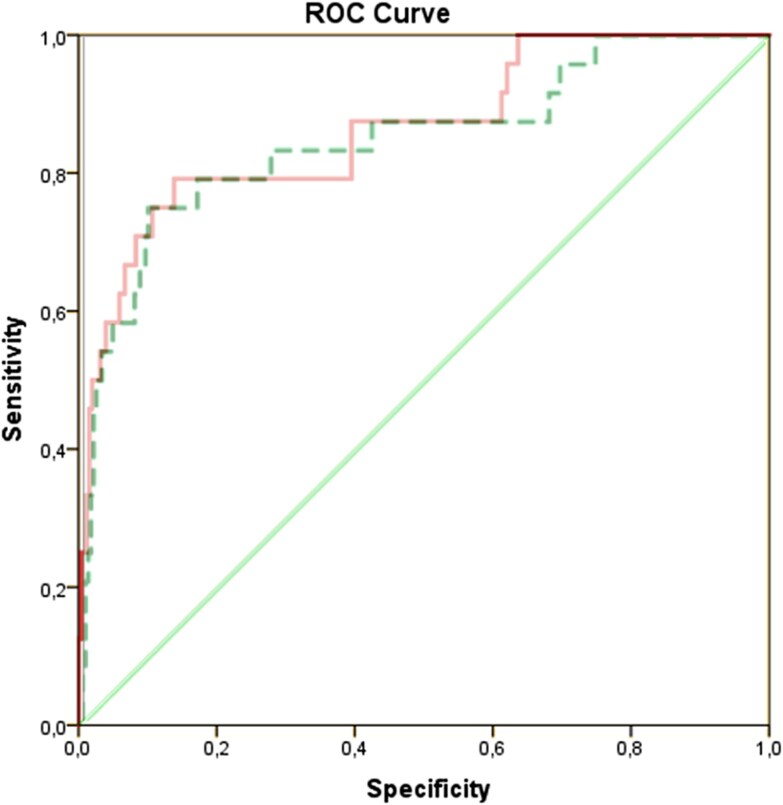
Receiver operating characteristic (ROC) curves for early all-cause mortality. ROC curves are shown for the clinical model including established risk factors (EuroSCORE, CPB time, urgent surgery, ECMO/inotropes weaning, mitral valve replacement) alone (blue line, AUC 0.854) and for the same model with the addition of TAPSE/PASP (red line, AUC 0.863). The modest improvement in discrimination suggests that TAPSE/PASP may provide incremental prognostic information, consistent with its independent association in multivariable logistic regression analyses. ROC curves reflect unadjusted discrimination; numerical differences should be interpreted cautiously.

The incremental value of TAPSE/PASP was explored by superimposing ROC curves for a clinical model including EuroSCORE, CPB time, urgent surgery, ECMO/inotrope weaning, and MV replacement, with and without TAPSE/PASP. Addition of TAPSE/PASP modestly increased the AUC from 0.854 to 0.863, supporting its potential to provide incremental prognostic information, consistent with its independent association in multivariable analysis (*Figure 5*).

**Figure 6.**
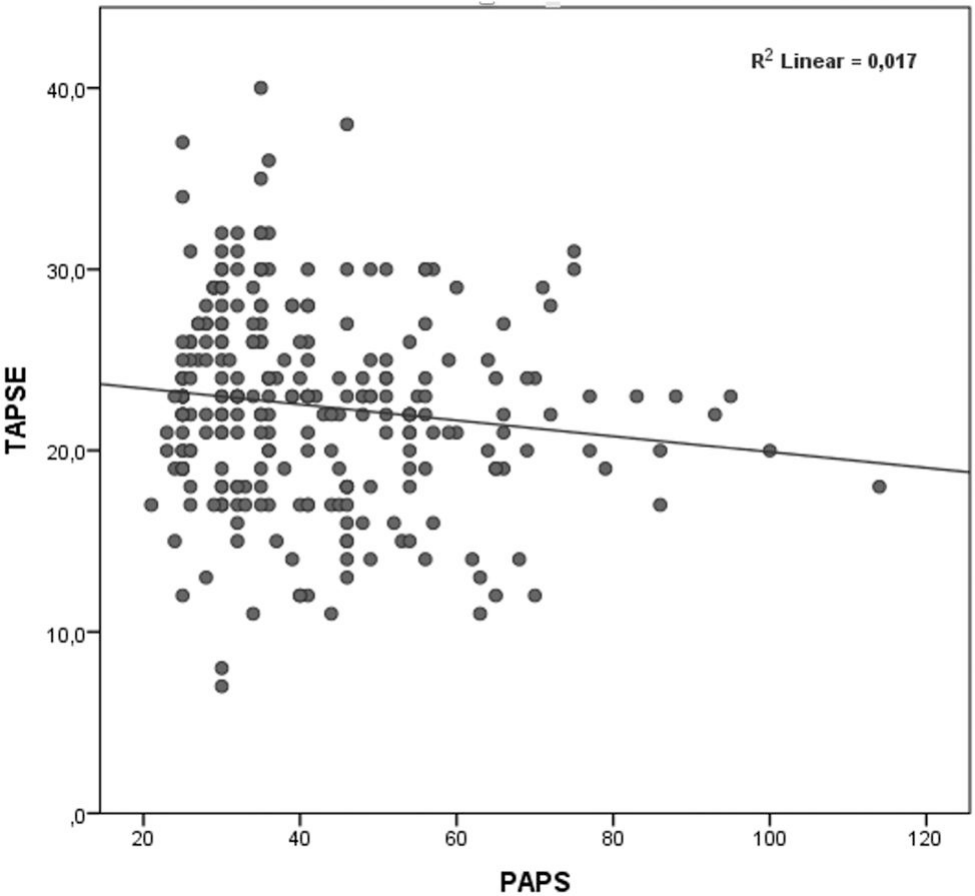
Scatterplot showing no correlation between TAPSE and PASP (Spearman’s ρ = 0.017).

We explored whether TAPSE or PASP contributed differentially to the predictive value of the TAPSE/PASP ratio. In univariable analysis, both TAPSE and PASP were individually associated with 30-day mortality. Previous studies, such as Butcher *et al*.’s, have demonstrated that elevated PASP (>50 mmHg) predicts mortality after MV surgery.^34^ Both TAPSE and PASP individually predicted 30-day mortality in univariable analysis, yet they were not correlated in our cohort, as shown by Spearman’s coefficient and the scatterplot of their distribution (*Figure 6*). This may, on one hand, reflect the inherent limitations of TAPSE as a measure that is dependent on pre-load and imaging angle. On the other hand, some patients may exhibit seemingly preserved TAPSE despite markedly elevated PASP, representing an ‘adapted’ RV that remains vulnerable to the acute hemodynamic stress of mitral surgery. This dissociation underscores the value of the TAPSE/PASP ratio: it could identify pre-operatively those at highest risk of early post-operative RVF and all-cause mortality, capturing vulnerability that would be missed by assessing TAPSE or PASP alone.

The pre-operative TAPSE/PASP ratio could serve as an adjunct to identify patients potentially at higher risk of early post-operative mortality or RHF, to complement multimodal assessment of RV function, and to guide hemodynamic optimization. The role of dynamic RV evaluation and contractile reserve assessment remains exploratory and should be validated in future prospective studies.

### Study limitations

This is a retrospective, single-centre study, with inherent design limitations. The cohort is heterogeneous but reflects a real-life population of patients undergoing surgery for severe MR.

We assessed TAPSE/PASP pre-operatively. Early post-operative measurements could be informative, yet TAPSE is less reliable post-operatively due to acute RV longitudinal dysfunction. Alternative RV–PA coupling indices could be considered, but TAPSE remains the most practical and widely used parameter in routine and urgent practice. PASP was not invasively validated in all patients.

The relatively small number of early deaths—both in the overall and isolated MV cohorts—necessitates cautious interpretation. Internal validation with bootstrapping was performed to enhance the robustness of the findings despite this limitation.

Finally, late outcomes are limited to survival status, without data on symptoms, quality of life, or ongoing therapy. Although no difference in late mortality was observed between coupled and uncoupled patients, other clinically relevant differences cannot be excluded.

## Conclusion

RV–PA uncoupling (TAPSE/PASP ≤0.30 mmHg) in patients undergoing surgery for severe MR was strongly associated with early all-cause and RHF mortality, as well as worse in-hospital outcomes. Its detection pre-operatively may help identify patients at higher surgical risk, particularly those with advanced PH, and could inform more tailored perioperative management strategies.

## Clinical perspectives

Pre-operative identification of patients in an RV–PA uncoupled state may aid surgical risk stratification and optimization of perioperative management in severe MR. This could be supported by a multimodal assessment of RV function, ideally complemented by dynamic testing, which represents a promising avenue for future research.

## Data Availability

The datasets and the materials used and analysed during the current study are available from the corresponding author on reasonable request.

## Lead Author Biography

Roxana Botea, MD, is a cardiologist at the Department of Cardiology and Cardiovascular Surgery, Rangueil University Hospital, Toulouse, France. Her clinical and research focus on valvular heart disease, infective endocarditis and complex cardiac surgery outcomes. She has authored several peer-reviewed publications in these fields, with particular involvement in multidisciplinary management of high-risk cardiovascular patients.
